# Supplementary material for: Efficacy of invasive laser acupuncture in treating chronic non-specific low back pain: A randomized controlled trial
Source: PLoS One. 2022 May 31;17(5):e0269282. doi: 10.1371/journal.pone.0269282 (PMC9154191; doi:10.1371/journal.pone.0269282)
Supplement: S1 Fig — (DOCX) [file pone.0269282.s001.docx]

**S1 Fig. The appearance and components of Ellise.**

| \| 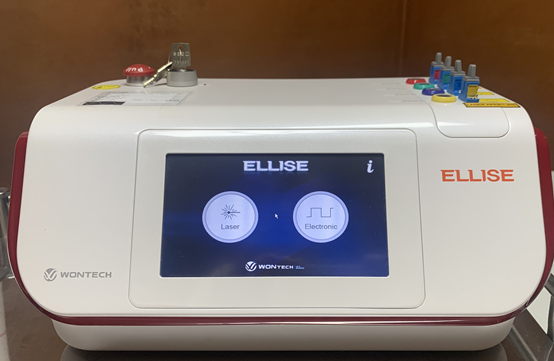 \| \| --- \| \| main body \| \| 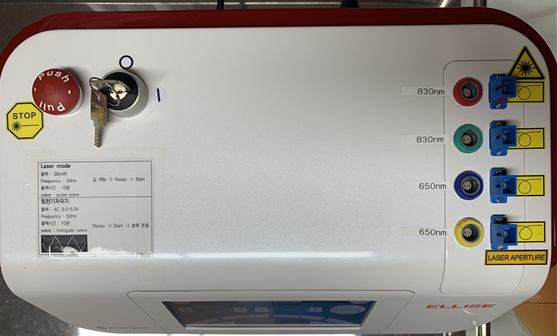 \| \| main body \| |  |
| --- | --- | --- | --- | --- | --- |
|  |  |
| \| 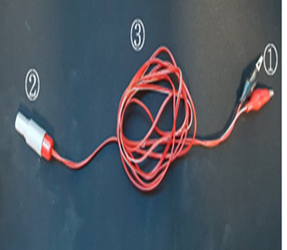 \| 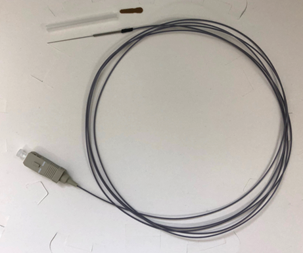 \| \| --- \| --- \| \| Electrical stimulus clip \| Acupuncture needle with optical fiber-coupled laser diode \| |  |
|  |  |
