# Supplementary material for: Efficacy of invasive laser acupuncture in treating chronic non-specific low back pain: A randomized controlled trial
Source: PLoS One. 2022 May 31;17(5):e0269282. doi: 10.1371/journal.pone.0269282 (PMC9154191; doi:10.1371/journal.pone.0269282)
Supplement: S2 Fig — (DOCX) [file pone.0269282.s002.docx]

S2 Fig. Invasive laser acupuncture procedure and electro acupuncture procedure

| 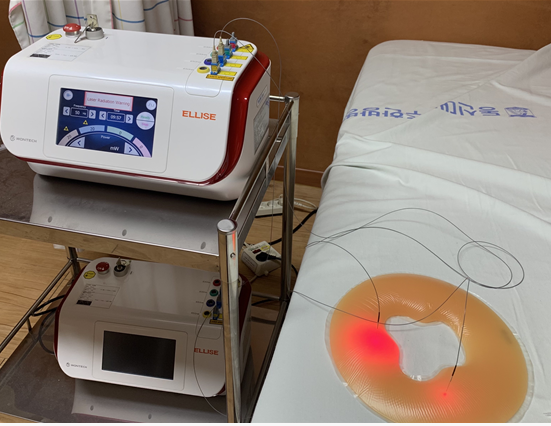 |
| --- |
| 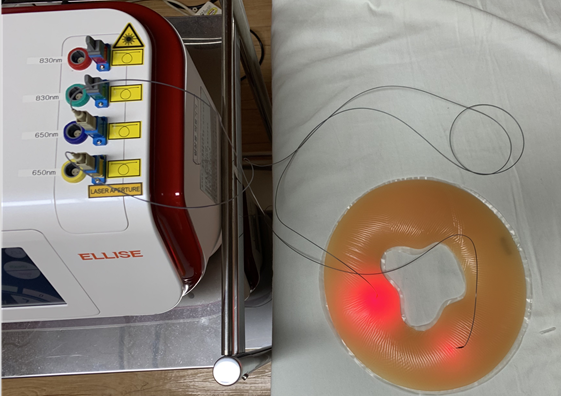 |
| Invasive laser acupuncture procedure |

| 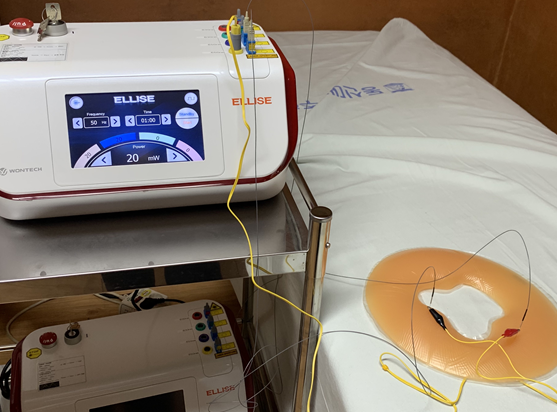 |
| --- |
| Electroacupuncture procedure |
