# Supplementary material for: Efficacy of invasive laser acupuncture in treating chronic non-specific low back pain: A randomized controlled trial
Source: PLoS One. 2022 May 31;17(5):e0269282. doi: 10.1371/journal.pone.0269282 (PMC9154191; doi:10.1371/journal.pone.0269282)
Supplement: S1 File — (DOCX) [file pone.0269282.s003.docx]

1. Baseline demographic characteristics

1.1. control group (n=15)

| Screening  No | Random  No | sex | age | Education  (Year) | Height  (cm) | Weight  (kg) | Drop out |
| --- | --- | --- | --- | --- | --- | --- | --- |
| EA-LA-S002 | EA-LA-R002 | M | 69 | 12 | 170.0 | 66.9 | No |
| EA-LA-S007 | EA-LA-R006 | F | 57 | 18 | 163.4 | 74.0 | No |
| EA-LA-S009 | EA-LA-R007 | F | 48 | 14 | 162.9 | 54.2 | No |
| EA-LA-S016 | EA-LA-R015 | F | 61 | 12 | 155.6 | 57.1 | No |
| EA-LA-S023 | EA-LA-R013 | F | 59 | 12 | 153.1 | 63.2 | No |
| EA-LA-S027 | EA-LA-R023 | F | 61 | 12 | 163.5 | 51.5 | No |
| EA-LA-S035 | EA-LA-R028 | F | 59 | 12 | 158.0 | 57.0 | Yes |
| EA-LA-S040 | EA-LA-R030 | F | 59 | 20 | 158.9 | 68.4 | No |
| EA-LA-S043 | EA-LA-R033 | F | 56 | 12 | 156.5 | 81.4 | Yes |
| EA-LA-S045 | EA-LA-R036 | F | 69 | 6 | 155.2 | 60.1 | No |
| EA-LA-S049 | EA-LA-R038 | F | 64 | 2 | 153.6 | 56.0 | Yes |
| EA-LA-S050 | EA-LA-R042 | F | 54 | 14 | 160.0 | 56.4 | No |
| EA-LA-S051 | EA-LA-R040 | M | 56 | 20 | 171.7 | 85.6 | No |
| EA-LA-S052 | EA-LA-R044 | F | 55 | 12 | 159.3 | 55.3 | No |
| EA-LA-S056 | EA-LA-R045 | F | 69 | 12 | 154.6 | 48.8 | No |

1.2. 650 group (n=15)

| screening  No | Random  No | sex | age | Education  (Year) | Heigh  t(cm) | Weigh  t(kg) | Drop out |
| --- | --- | --- | --- | --- | --- | --- | --- |
| EA-LA-S001 | EA-LA-R001 | F | 64 | 9 | 159.1 | 62.2 | No |
| EA-LA-S004 | EA-LA-R004 | F | 65 | 12 | 161.9 | 57.9 | No |
| EA-LA-S008 | EA-LA-R005 | F | 56 | 20 | 158.5 | 63.6 | No |
| EA-LA-S013 | EA-LA-R011 | M | 57 | 12 | 164.1 | 72.1 | No |
| EA-LA-S014 | EA-LA-R009 | F | 59 | 12 | 166.1 | 80.6 | No |
| EA-LA-S017 | EA-LA-R010 | M | 59 | 16 | 160.7 | 65.0 | No |
| EA-LA-S021 | EA-LA-R012 | M | 66 | 12 | 169.1 | 67.5 | No |
| EA-LA-S026 | EA-LA-R014 | F | 57 | 9 | 161.5 | 71.4 | No |
| EA-LA-S028 | EA-LA-R020 | F | 58 | 12 | 157.8 | 58.5 | No |
| EA-LA-S030 | EA-LA-R021 | F | 59 | 6 | 155.1 | 82.3 | No |
| EA-LA-S031 | EA-LA-R024 | F | 41 | 16 | 152.1 | 80.5 | No |
| EA-LA-S036 | EA-LA-R031 | F | 57 | 12 | 157.8 | 57.3 | No |
| EA-LA-S039 | EA-LA-R032 | M | 60 | 12 | 168.4 | 89.4 | No |
| EA-LA-S041 | EA-LA-R029 | M | 65 | 14 | 171.8 | 73.6 | No |
| EA-LA-S055 | EA-LA-R043 | M | 54 | 12 | 171.8 | 74.6 | No |

1.3. 830 group (n=15)

| Screening  No | Random  No | sex | age | Education  (Year) | Heigh  t(cm) | Weight  (kg) | Drop out |
| --- | --- | --- | --- | --- | --- | --- | --- |
| EA-LA-S003 | EA-LA-R003 | M | 68 | 12 | 167.9 | 70.6 | No |
| EA-LA-S011 | EA-LA-R008 | F | 34 | 16 | 161.4 | 52.1 | No |
| EA-LA-S015 | EA-LA-R016 | M | 59 | 20 | 164.9 | 78.2 | No |
| EA-LA-S018 | EA-LA-R017 | M | 66 | 9 | 172.8 | 68.6 | No |
| EA-LA-S020 | EA-LA-R018 | F | 55 | 12 | 157.1 | 51.0 | No |
| EA-LA-S029 | EA-LA-R019 | M | 31 | 14 | 171.5 | 65.9 | No |
| EA-LA-S032 | EA-LA-R022 | F | 59 | 12 | 159.1 | 73.1 | Yes |
| EA-LA-S033 | EA-LA-R026 | F | 55 | 12 | 160.8 | 73.5 | No |
| EA-LA-S034 | EA-LA-R025 | M | 68 | 12 | 156.4 | 59.0 | No |
| EA-LA-S037 | EA-LA-R027 | F | 36 | 15 | 160.3 | 51.4 | No |
| EA-LA-S038 | EA-LA-R034 | M | 52 | 20 | 166.7 | 90.2 | No |
| EA-LA-S046 | EA-LA-R039 | F | 52 | 12 | 160.0 | 68.0 | No |
| EA-LA-S047 | EA-LA-R035 | F | 69 | 12 | 153.2 | 56.8 | No |
| EA-LA-S048 | EA-LA-R037 | F | 57 | 6 | 151.3 | 59.3 | Yes |
| EA-LA-S054 | EA-LA-R041 | F | 55 | 16 | 160.1 | 60.7 | No |

2. Efficacy Outcomes

2.1. VAS scores in control group (n=15)

| Random No | Week 0 | Week 4 | Week 8 |
| --- | --- | --- | --- |
| EA-LA-R002 | 33 | 5 | 9 |
| EA-LA-R006 | 54 | 2 | 1 |
| EA-LA-R007 | 57 | 22 | 27 |
| EA-LA-R015 | 50 | 41 | 50 |
| EA-LA-R013 | 47 | 13 | 6 |
| EA-LA-R023 | 68 | 75 | 44 |
| EA-LA-R028 | 61 | 40 | 40 |
| EA-LA-R030 | 45 | 44 | 44 |
| EA-LA-R033 | 45 | 45 | 45 |
| EA-LA-R036 | 43 | 50 | 59 |
| EA-LA-R038 | 44 | 44 | 44 |
| EA-LA-R042 | 39 | 34 | 18 |
| EA-LA-R040 | 59 | 48 | 63 |
| EA-LA-R044 | 53 | 33 | 9 |
| EA-LA-R045 | 59 | 47 | 53 |

2.2. VAS scores in 650 group (n=15)

| Random No | Week 0 | Week 4 | Week 8 |
| --- | --- | --- | --- |
| EA-LA-R001 | 96 | 3 | 70 |
| EA-LA-R004 | 52 | 26 | 72 |
| EA-LA-R005 | 69 | 1 | 0 |
| EA-LA-R011 | 58 | 34 | 30 |
| EA-LA-R009 | 67 | 27 | 9 |
| EA-LA-R010 | 33 | 22 | 10 |
| EA-LA-R012 | 32 | 13 | 13 |
| EA-LA-R014 | 70 | 37 | 5 |
| EA-LA-R020 | 48 | 22 | 12 |
| EA-LA-R021 | 52 | 20 | 4 |
| EA-LA-R024 | 75 | 41 | 50 |
| EA-LA-R031 | 39 | 17 | 13 |
| EA-LA-R032 | 40 | 30 | 34 |
| EA-LA-R029 | 47 | 32 | 25 |
| EA-LA-R043 | 48 | 22 | 51 |

2.3. VAS scores in 830 group (n=15)

| Random No | Week 0 | Week 4 | Week 8 |
| --- | --- | --- | --- |
| EA-LA-R003 | 54 | 34 | 25 |
| EA-LA-R008 | 48 | 36 | 34 |
| EA-LA-R016 | 49 | 30 | 10 |
| EA-LA-R017 | 54 | 13 | 27 |
| EA-LA-R018 | 57 | 56 | 47 |
| EA-LA-R019 | 21 | 6 | 1 |
| EA-LA-R022 | 50 | 50 | 50 |
| EA-LA-R026 | 40 | 20 | 18 |
| EA-LA-R025 | 41 | 26 | 10 |
| EA-LA-R027 | 69 | 27 | 4 |
| EA-LA-R034 | 50 | 41 | 42 |
| EA-LA-R039 | 54 | 32 | 28 |
| EA-LA-R035 | 43 | 5 | 12 |
| EA-LA-R037 | 38 | 38 | 38 |
| EA-LA-R041 | 76 | 15 | 33 |

2.4. ODI scores in control group (n=15)

| Random No | Week 0 | Week 4 | Week 8 |
| --- | --- | --- | --- |
| EA-LA-R002 | 10 | 6 | 6 |
| EA-LA-R006 | 8 | 5 | 3 |
| EA-LA-R007 | 8 | 7 | 7 |
| EA-LA-R015 | 8 | 5 | 8 |
| EA-LA-R013 | 9 | 3 | 2 |
| EA-LA-R023 | 13 | 21 | 17 |
| EA-LA-R028 | 15 | 11 | 11 |
| EA-LA-R030 | 10 | 11 | 14 |
| EA-LA-R033 | 11 | 11 | 11 |
| EA-LA-R036 | 7 | 9 | 6 |
| EA-LA-R038 | 5 | 5 | 5 |
| EA-LA-R042 | 6 | 7 | 8 |
| EA-LA-R040 | 25 | 21 | 29 |
| EA-LA-R044 | 6 | 0 | 0 |
| EA-LA-R045 | 11 | 12 | 8 |

2.5. ODI scores in 650 group (n=15)

| Random No | Week 0 | Week 4 | Week 8 |
| --- | --- | --- | --- |
| EA-LA-R001 | 20 | 7 | 13 |
| EA-LA-R004 | 19 | 8 | 11 |
| EA-LA-R005 | 17 | 0 | 1 |
| EA-LA-R011 | 12 | 7 | 11 |
| EA-LA-R009 | 10 | 7 | 3 |
| EA-LA-R010 | 12 | 7 | 4 |
| EA-LA-R012 | 8 | 7 | 11 |
| EA-LA-R014 | 12 | 12 | 3 |
| EA-LA-R020 | 9 | 7 | 5 |
| EA-LA-R021 | 10 | 2 | 2 |
| EA-LA-R024 | 24 | 14 | 20 |
| EA-LA-R031 | 8 | 7 | 6 |
| EA-LA-R032 | 8 | 7 | 5 |
| EA-LA-R029 | 11 | 5 | 5 |
| EA-LA-R043 | 11 | 8 | 9 |

2.6. ODI scores in 830 group (n=15)

| Random No | Week 0 | Week 4 | Week 8 |
| --- | --- | --- | --- |
| EA-LA-R003 | 7 | 6 | 5 |
| EA-LA-R008 | 8 | 5 | 4 |
| EA-LA-R016 | 9 | 2 | 7 |
| EA-LA-R017 | 15 | 1 | 5 |
| EA-LA-R018 | 16 | 11 | 12 |
| EA-LA-R019 | 12 | 5 | 0 |
| EA-LA-R022 | 7 | 7 | 7 |
| EA-LA-R026 | 11 | 7 | 6 |
| EA-LA-R025 | 8 | 3 | 0 |
| EA-LA-R027 | 13 | 4 | 2 |
| EA-LA-R034 | 11 | 9 | 6 |
| EA-LA-R039 | 10 | 3 | 6 |
| EA-LA-R035 | 4 | 3 | 5 |
| EA-LA-R037 | 14 | 14 | 14 |
| EA-LA-R041 | 13 | 6 | 11 |

2.7. EQ-5D-5L scores in control group (n=15)

| Random No | Week 0 | Week 4 | Week 8 |
| --- | --- | --- | --- |
| EA-LA-R002 | 9 | 7 | 6 |
| EA-LA-R006 | 7 | 5 | 5 |
| EA-LA-R007 | 6 | 5 | 5 |
| EA-LA-R015 | 10 | 6 | 9 |
| EA-LA-R013 | 8 | 5 | 5 |
| EA-LA-R023 | 9 | 14 | 9 |
| EA-LA-R028 | 11 | 9 | 9 |
| EA-LA-R030 | 6 | 8 | 10 |
| EA-LA-R033 | 6 | 6 | 6 |
| EA-LA-R036 | 6 | 8 | 8 |
| EA-LA-R038 | 9 | 9 | 9 |
| EA-LA-R042 | 7 | 5 | 6 |
| EA-LA-R040 | 11 | 10 | 20 |
| EA-LA-R044 | 6 | 5 | 5 |
| EA-LA-R045 | 8 | 11 | 10 |

2.8. EQ-5D-5L scores in 650 group (n=15)

| Random No | Week 0 | Week 4 | Week 8 |
| --- | --- | --- | --- |
| EA-LA-R001 | 13 | 8 | 9 |
| EA-LA-R004 | 11 | 5 | 9 |
| EA-LA-R005 | 14 | 5 | 5 |
| EA-LA-R011 | 10 | 7 | 7 |
| EA-LA-R009 | 10 | 6 | 8 |
| EA-LA-R010 | 8 | 5 | 5 |
| EA-LA-R012 | 7 | 8 | 8 |
| EA-LA-R014 | 10 | 9 | 5 |
| EA-LA-R020 | 7 | 6 | 6 |
| EA-LA-R021 | 9 | 7 | 5 |
| EA-LA-R024 | 11 | 11 | 11 |
| EA-LA-R031 | 9 | 7 | 6 |
| EA-LA-R032 | 9 | 7 | 7 |
| EA-LA-R029 | 8 | 8 | 7 |
| EA-LA-R043 | 7 | 7 | 9 |

2.9. EQ-5D-5L scores in 830 group (n=15)

| Random No | Week 0 | Week 4 | Week 8 |
| --- | --- | --- | --- |
| EA-LA-R003 | 8 | 6 | 6 |
| EA-LA-R008 | 7 | 7 | 7 |
| EA-LA-R016 | 7 | 6 | 6 |
| EA-LA-R017 | 9 | 7 | 7 |
| EA-LA-R018 | 11 | 8 | 7 |
| EA-LA-R019 | 5 | 5 | 5 |
| EA-LA-R022 | 7 | 7 | 7 |
| EA-LA-R026 | 9 | 5 | 9 |
| EA-LA-R025 | 9 | 5 | 5 |
| EA-LA-R027 | 8 | 7 | 5 |
| EA-LA-R034 | 8 | 10 | 6 |
| EA-LA-R039 | 7 | 5 | 6 |
| EA-LA-R035 | 7 | 6 | 7 |
| EA-LA-R037 | 10 | 10 | 10 |
| EA-LA-R041 | 12 | 7 | 8 |

3. Safety Evaluation

3.1. Vital sign of control group (n=15)

| Random No | Week 0 | | | | | Week 4 | | | | |
| --- | --- | --- | --- | --- | --- | --- | --- | --- | --- | --- |
|  | Systoric BP | Diastoric BP | Pulse | Respiration | Temperature | Systoric BP | Diastoric BP | Pulse | Respiration | Temperature |
| R002 | 130 | 90 | 80 | 20 | 36.0 | 119 | 77 | 67 | 20 | 36.0 |
| R006 | 130 | 74 | 77 | 20 | 36.8 | 130 | 80 | 78 | 20 | 36.5 |
| R007 | 112 | 67 | 64 | 20 | 36.7 | 99 | 59 | 70 | 20 | 37.1 |
| R015 | 106 | 73 | 67 | 20 | 36.5 | 104 | 75 | 73 | 20 | 36.2 |
| R013 | 121 | 79 | 55 | 20 | 36.0 | 118 | 78 | 54 | 20 | 36.0 |
| R023 | 115 | 75 | 76 | 20 | 36.7 | 114 | 70 | 80 | 20 | 36.8 |
| R028 | 151 | 92 | 55 | 20 | 36.4 | 148 | 88 | 60 | 20 | 36.7 |
| R030 | 125 | 88 | 89 | 20 | 36.8 | 137 | 97 | 78 | 20 | 36.4 |
| R033 | 120 | 87 | 85 | 20 | 36.5 | - | - | - | - | - |
| R036 | 129 | 80 | 75 | 20 | 37.0 | 143 | 85 | 76 | 20 | 36.6 |
| R038 | 118 | 97 | 100 | 20 | 37.0 | - | - | - | - | - |
| R042 | 134 | 82 | 57 | 20 | 36.7 | 128 | 88 | 60 | 20 | 36.6 |
| R040 | 127 | 80 | 80 | 20 | 36.6 | 127 | 97 | 99 | 20 | 36.0 |
| R044 | 107 | 77 | 79 | 20 | 36.5 | 104 | 78 | 75 | 20 | 36.5 |
| R045 | 137 | 91 | 65 | 20 | 36.7 | 113 | 71 | 67 | 20 | 36.9 |

3.2. Vital sign of 650 group (n=15)

| Random No | Week 0 | | | | | Week 4 | | | | |
| --- | --- | --- | --- | --- | --- | --- | --- | --- | --- | --- |
|  | Systoric BP | Diastoric BP | Pulse | Respiration | Temperature | Systoric BP | Diastoric BP | Pulse | Respiration | Temperature |
| R001 | 109 | 82 | 66 | 20 | 36.5 | 119 | 77 | 67 | 20 | 36.0 |
| R004 | 117 | 84 | 82 | 20 | 37.0 | 103 | 64 | 76 | 20 | 37.0 |
| R005 | 122 | 70 | 71 | 20 | 36.8 | 128 | 83 | 71 | 20 | 36.8 |
| R011 | 141 | 86 | 76 | 20 | 37.2 | 150 | 92 | 71 | 20 | 36.5 |
| R009 | 129 | 94 | 80 | 22 | 36.8 | 120 | 86 | 103 | 20 | 37.0 |
| R010 | 115 | 78 | 75 | 20 | 36.8 | 107 | 73 | 73 | 20 | 36.2 |
| R012 | 120 | 66 | 80 | 20 | 36.7 | 105 | 65 | 83 | 20 | 36.4 |
| R014 | 123 | 71 | 75 | 20 | 37.0 | 112 | 76 | 73 | 20 | 37.0 |
| R020 | 121 | 74 | 77 | 20 | 37.1 | 112 | 76 | 79 | 20 | 36.9 |
| R021 | 131 | 78 | 77 | 20 | 37.0 | 141 | 87 | 69 | 20 | 36.9 |
| R024 | 112 | 81 | 80 | 20 | 37.0 | 122 | 88 | 83 | 20 | 36.3 |
| R031 | 113 | 81 | 64 | 20 | 36.5 | 116 | 82 | 60 | 20 | 36.1 |
| R032 | 143 | 71 | 81 | 20 | 36.7 | 127 | 71 | 82 | 20 | 36.5 |
| R029 | 140 | 88 | 70 | 20 | 36.6 | 129 | 81 | 88 | 20 | 36.9 |
| R043 | 148 | 95 | 81 | 20 | 36.9 | 162 | 114 | 79 | 20 | 37.1 |

3.3. Vital sign of 830 group (n=15)

| Random No | Week 0 | | | | | Week 4 | | | | |
| --- | --- | --- | --- | --- | --- | --- | --- | --- | --- | --- |
|  | Systoric BP | Diastoric BP | Pulse | Respiration | Temperature | Systoric BP | Diastoric BP | Pulse | Respiration | Temperature |
| R003 | 114 | 80 | 65 | 18 | 36.8 | 112 | 81 | 68 | 20 | 36.8 |
| R008 | 107 | 78 | 72 | 20 | 36.8 | 110 | 69 | 97 | 20 | 37.2 |
| R016 | 136 | 95 | 68 | 20 | 36.6 | 138 | 86 | 85 | 20 | 36.7 |
| R017 | 119 | 72 | 87 | 20 | 36.6 | 154 | 88 | 78 | 20 | 36.4 |
| R018 | 101 | 72 | 87 | 22 | 36.5 | 93 | 70 | 88 | 20 | 36.4 |
| R019 | 129 | 84 | 96 | 20 | 36.5 | 139 | 87 | 78 | 20 | 37.1 |
| R022 | 132 | 89 | 82 | 20 | 37.3 | - | - | - | - | - |
| R026 | 124 | 78 | 61 | 20 | 36.7 | 107 | 71 | 63 | 20 | 36.8 |
| R025 | 129 | 79 | 53 | 20 | 36.6 | 136 | 88 | 56 | 20 | 36.4 |
| R027 | 107 | 79 | 75 | 20 | 36.5 | 112 | 70 | 80 | 20 | 36.6 |
| R034 | 137 | 94 | 83 | 20 | 36.3 | 126 | 82 | 73 | 20 | 36.2 |
| R039 | 111 | 86 | 81 | 20 | 36.4 | 112 | 75 | 70 | 20 | 36.5 |
| R035 | 137 | 88 | 79 | 20 | 37.2 | 136 | 87 | 82 | 20 | 36.9 |
| R037 | 117 | 75 | 66 | 20 | 36.6 | - | - | - | - | - |
| R041 | 111 | 72 | 65 | 20 | 36.5 | 109 | 60 | 63 | 20 | 36.8 |

3.4. Adverse Events related to intervention.

| Group | Random No. | symptom | occurrence date | improvement date | results | severity | drop out | Treatment |
| --- | --- | --- | --- | --- | --- | --- | --- | --- |
| control | R006 | hematoma | June 19, 2020 | June 25, 2020 | recovered | mild | No | None |
| control | R040 | nausea | August 25, 2020 | August 27, 2020 | recovered | mild | No | None |
