# Supplementary material for: Efficacy of invasive laser acupuncture in treating chronic non-specific low back pain: A randomized controlled trial
Source: PLoS One. 2022 May 31;17(5):e0269282. doi: 10.1371/journal.pone.0269282 (PMC9154191; doi:10.1371/journal.pone.0269282)
Supplement: Attachment — Submitted filename: (second) response to reviewer-PLOS ONE.docx [file pone.0269282.s007.docx]

We are very grateful for the constructive comments and the opportunity to revise our manuscript.

Point-by-point responses to the reviewers’ comments are provided below. The corresponding changes have been highlighted in the revised manuscript with track changes.

6. Review Comments to the Author

Please use the space provided to explain your answers to the questions above. You may also include additional comments for the author, including concerns about dual publication, research ethics, or publication ethics. (Please upload your review as an attachment if it exceeds 20,000 characters)

Reviewer #2: The paper reads much clearer after the revision.
A few minor changes are required:
page 11, line 220: the first word 'efficacy' should be 'effectiveness':
"Because our study was a pilot study, the sample size was insufficient to determine the efficacy of ILA for CNLBP.".
**Response.** Thank you for your comments. In accordance with your suggestion, We revised ‘efficacy’ to ‘effectiveness’

Table 3 needs editing; some words are cut in half. Same as Table 7.

**Response.** Thank you for your comments. In accordance with your suggestion,we edited Table 3 and 7.

Table 6, there should be a gap between 'group' and (n=15).

**Response.** Thank you for your comments. In accordance with your suggestion,we edited Table 6.

In figure 1, the first letter should be F, not f.

**Response.** Thank you for your comments. In accordance with your suggestion,we revised the figure 1.
